# A Novel GH Family 20 β-N-acetylhexosaminidase With Both Chitosanase and Chitinase Activity From *Aspergillus oryzae*

**DOI:** 10.3389/fmolb.2021.684086

**Published:** 2021-05-19

**Authors:** Tianle Qu, Chunyue Zhang, Zhen Qin, Liqiang Fan, Lihua Jiang, Liming Zhao

**Affiliations:** ^1^ School of Biotechnology, State Key Laboratory of Bioreactor Engineering, R&D Center of Separation and Extraction Technology in Fermentation Industry, East China University of Science and Technology, Shanghai, China; ^2^ School of Life Science, Shanghai University, Shanghai, China; ^3^ Shanghai Collaborative Innovation Center for Biomanufacturing Technology (SCICBT), Shanghai, China

**Keywords:** β-N-acetylhexosaminidase, aminooligosaccharide, glycoside hydrolase family 20, chitosan, chitin

## Abstract

Aminooligosaccharides possess various biological activities and can exploit wide applications in food, pharmaceutical and cosmetic industries. Commercial aminooligosaccharides are often prepared by the hydrolysis of chitin and chitosan. In this study, a novel GH family 20 β-N-acetylhexosaminidases gene named AoNagase was cloned from *Aspergillus oryzae* and expressed in *Pichia pastoris*. The purified AoNagase had maximal activity at pH 5.5 and 65°C. It exhibited good pH stability in the range of pH 6.0–7.5 and at temperatures below 50°C. AoNagase was capable of hydrolyzing not only colloidal chitosan (508.26 U/mg) but also chitin (29.78 U/mg). The kinetic parameters (*K*_*m*_ and *V*_*max*_) of AoNagase were 1.51 mM, 1106.02 U/mg for chitosan and 0.41 mM, 40.31 U/mg for colloidal chitin. To our knowledge, AoNagase is the first GH family 20 β-N-acetylhexosaminidase capable of hydrolyzing both chitosan and chitin. AoNagase is an endo-type β-N-acetylhexosaminidases and can potentially be used for the manufacturing of aminooligosaccharides.

## Introduction

Chitin, the second most abundant natural polysaccharide next to cellulose in nature (Muzzarelli 2010), is an insoluble linear aminopolysaccharide comprising of 1,4-β-linked N-acetyl-D-glucosamine (GlcNAc) (Kumari et al., 2017). Chitin exist in fungi, coralline algae, sponges, mollusks, insects and crustacean shells (Kim et al., 2017; Srinivasan et al., 2018; Crini 2019). Chitosan is the deacetylation product of chitin (Muzzarelli 2010). Chitin oligosaccharides (ChOS) and chitooligosaccharides (COS), collectively referred to as aminooligosaccharides, are the hydrolysis products of chitin and chitosan with degrees of polymerization (DPs) lower than 10 (Aam et al., 2010; Qin and Zhao 2019). Due to their nontoxic, superior solubility properties and various physiological functions, aminooligosaccharides have broad application prospects in the field of functional food, cosmetics, medicaments and agriculture (Das et al., 2015; Zou et al., 2016; Kritchenkov et al., 2021). Thus, the large-scale production of aminooligosaccharides has attracted increasing attention.

Various techniques have thus far been used for the production of aminooligosaccharides from chitinous biomass, including chemical (Kazami et al., 2015), physical (Ajavakom et al., 2012), and enzymatic methods (Tae et al., 2018). Compared with the high-polluting chemical degradation and the high-energy-cost physical degradation, the enzymatic method is superior due to its mild reaction condition, high yield, low side reaction and minor pollution (Joseph et al., 2021). Therefore, chitinase (EC3.2.1.14/EC3.2.1.52) and chitosanase (EC3.2.1.132), the essential glycosyl hydrolases for the biotransformation of chitin and chitosan into aminooligosaccharides, become one of the research hotspots.

Chitosanases can degrade the β-1,4-glycoside bond in chitosan. Several chitosanases have been characterized (Shinya and Fukamizo 2017), they mainly belong to GH 3, GH 5, GH 7, GH 8, GH 46, GH 75, and GH 80 in the Carbohydrate-Active EnZYmes database (CAZy:http://www.cazy.org/). Among them, GH family 46 chitosanases are characterized with their excellent catalytic capacity and their potentials to be applied in the production of COS (Viens et al., 2015; Luo et al., 2020). Compared with chitosanases, chitinases are much less reported. According to the cleavage type, chitinases are divided into endo-type chitinases (EC3.2.1.14) and exo-type chitinases (EC3.2.1.52, also called β-N-acetylhexosaminidases) (Beygmoradi et al., 2018; Schmitz et al., 2019). In the CAZy database, endo-type chitinases mainly exit in GH 18 and GH 19, while exo-type chitinases mainly exit in GH 18 and GH 20. Most of the enzymes in GH family 20 are β-N-acetylhexosaminidases and are specific for the cleavage of both β-GlcNAc and β-N-acetylgalactosamine (β-GalNAc) units in a variety of substrates, such as chitin, ChOS, glycosphingolipids and other glycoconjugates (Slámová and Bojarová 2017; Liu et al., 2018). Owing to the ability to hydrolyze a wide variety of substrates, and the broad resources including bacteria, fungi, insects, plants, vertebrates and human (Intra et al., 2008; Wang et al., 2018), chitinases in GH 20 family have been selected in this studied.

*Aspergillus oryzae*, a ‘generally regarded as safe’ (GRAS) species with clear genetic background, plays a significant role in soybean fermentation (Contesini et al., 2010; Ding et al., 2019). The whole genome of *A. oryzae* RIB40 has been sequenced in 2005, and the sequence annotations indicate that *A. oryzae* can produce a wide range of useful enzymes (Machida et al., 2005; Zhao et al., 2012; Cerqueira et al., 2014). In the CAZy database, a GH family 20 gene from *A. oryzae* (GenBank: BAE58709.1) was inferred as β-N-acetylhexosaminidase by homology, which means it has the potential to hydrolyze chitin and ChOS (The UniProt, Consortium., 2019; Morgat et al., 2020). Thus, in this work, the gene aforesaid was cloned and expressed in *Pichia pastoris*. After purification, the properties of the recombinant enzyme (named AoNagase) were characterized. Unexpectedly, AoNagase showed both chitosanases and chitinase activity, and its chitosanase activity is much higher than that of chitinase. To our knowledge, this is the first enzyme found in GH family 20 that has the ability to degrade chitosan.

## Methods

### Strains, Plasmids and Chemicals

*A. oryzae* was isolated from spores of koji soy sauce (Bright Dairy, Shanghai, China) and cultivated in potato dextrose broth (PDB) agar plate (Ueki et al., 1994). *Escherichia coli* DH5α cells obtained from Cwbio (Jiangsu, China), were cultivated in Luria-Bertani (LB) broth medium or agar plate. *Pichia pastoris* GS115 cells were cultivated in minimal dextrose (MD) agar plate or buffered minimal glycerol complex (BMGY) medium and induced in buffered minimal methanol complex (BMMY) medium. The pPIC9k vector was used for the cloning and expression of target gene. Pfu DNA polymerase was purchased from Sangon Biotech (Shanghai, China). Restriction endonucleases and T4 DNA ligase were obtained from TaKaRa (Beijing, China). The reverse transcription kit was purchased from Vazyme Biotech (Jiangsu, China). The *p*-nitrophenyl-N-acetyl-β-d-glucosaminide (*p*NP-GlcNAc) and TRIzol were purchased from Yuanye Bio-Technology (Shanghai, China). Chitosan (degree of deacetylation ≥90%), chitin and D-glucosamine hydrochloride were purchased from Aladdin (Shanghai, China), the structures of chitosan and chitin are shown in the Supplementary Figure 1. GlcNAc was purchased from Macklin (Shanghai, China). COS monomers (DP 2–6), the mixed standards of (GlcN)_1–6_ and (GlcNAc)_1–3_ were all prepared in our laboratory (Qin et al., 2018b; Chen et al., 2019). All other chemicals were commercially available and of analytical grade.

### Sequence Analysis of AoNagase

CAZy (http://www.cazy.org/) was used to screen potential target genes of GH family 20. BLASTp (https://blast.ncbi.nlm.nih.gov) was used to compare the homology of the target genomic sequences in the NCBI database. SignalP 5.0 Server (http://www.cbs.dtu.dk/services/SignalP/) was used to predicte N-terminal signal peptide. ExPASy (http://www.expasy.org/tools) was used to predicte molecular weight (MW) and isoelectric point (pI) (Artimo et al., 2012). MEGA (Version 7.0, Home Page, China) was used to generate evolutionary trees by the method of neighbor-joining (Saitou and Nei 1987; Kumar et al., 2016). SWISS-MODEL Repository (https://swissmodel.expasy.org/) was used to generate 3D protein structure models with annotated by the SWISS-MODEL homology-modelling pipeline (Bienert et al., 2017; Waterhouse et al., 2018).

### Preparation of cDNA

The total RNA of *A. oryzae* was extracted using TRIzol reagent as described by Chomczynski (2006) with minor modifications (Bo et al., 2020). Individual bacterial colonies of *A. oryza* were isolated and incubated in PDB medium at 30°C with shaking at 150 rpm for 72 h, and then moderate drained mycelia was frozen by liquid nitrogen and powdered using mortar and pestle. The ground powder was moved into an eppendorf tube where 1 ml TRIzol was added. The mixture was kept at room temperature (RT) for 5 min and then 0.2 ml chloroform was added. After vortexing for 15 s, the mixture was kept at RT for 3 min, followed by centrifuged at 12,000 rpm at 4°C for 15 min. The supernatant was transferred into a new centrifuge tube and this step was repeated twice. Then 0.5 ml isopropanol was added to the supernatant and kept at RT for 10 min. After centrifuged at 12,000 rpm at 4°C for 10 min, the supernatant was discarded and the precipitate was washed with 75% cold ethanol, and the RNA pellet was dried at RT for 20 min. Finally, the RNA pellet was dissolved in 50–100 μl RNase free water at 65°C.

The cDNA of *A. oryzae* was obtained by reverse transcription from the total RNA aforementioned. Total RNA and cDNA were all stored at −80°C before further use.

### Cloning and Expression of AoNagase in *P. pastoris*

To express AoNagase in *P. pastoris*, the target gene was amplified from the *A. oryzae* cDNA. Restriction endonucleases *SnaB* I and *Not* I sites (underlined) were added to the forward and reverse primers (AoNagase-up, ATT​TAA​CAC​GTAC​GTAATG​TGC​GCG​TTT​CTG​TCC​TT; AoNagase-down, ATT​AAA​TATGCG​GCC​GCTCA​GTC​AGC​ACT​CCC​ACG​TAG), respectively.

The PCR product was gel-purified, digested with *SnaB* I and *Not* I, and cloned into the corresponding sites of pPIC9k. The recombinant plasmid was transformed into *E. coli* DH5α competent cells. The positive DH5α recombinants were identified by PCR and DNA sequencing (Sangon Biotech, Shanghai, China) and transformed into *P. pastoris* GS115 competent cells for expression. Subsequently, a positive GS115 transformant was screened and cultivated in MD agar plate with gradient concentrations of Geneticin (G418) for obtaining multicopy recombinants. The positive multicopy transformants were incubated in BMGY medium at 30°C with shaking at 220 rpm until the OD_600_ reached 2–6. And then, the mixtures were centrifuged at 6000 rpm at 4°C for 5 min, the supernatant was discarded, the precipitate cells were collected and inoculated into BMMY medium until the OD_600_ reached 1. The cultures were cultured in the same conditions as induction and expression. As an inducer, methanol was added to the solution every 24 h to a final concentration of 0.5% for continuous 5 days. The cultures (100 ml) were centrifuged at 10,000 rpm at 4°C for 10 min after induction. The supernatant was first concentrated by centrifuging at 5,000 rpm for 30 min at 4°C with ultrafiltration membranes (10 kDa cut-off), after which the concentrate solution obtained was collected as crude enzyme.

### Purification of Recombinant AoNagase

The crude enzyme was dialysed (10 kDa cut-off) against Tris-HCl buffer (20 mM, pH 7.9) containing150 mM NaCl at 4°C for 16 h. The Q-Sepharose fast flow ion exchange column (1 × 5 cm) was equilibrated with the same Tris-HCl buffer, and then 10 ml dialyzed crude enzyme was loaded on the column with the flow rate of 0.5 ml/min. The unbound proteins were eluted with equilibrium solution firstly, the bound proteins were gradiently eluted with different concentrations of NaCl solution (150 mM–500 mM NaCl, 20 mM Tris-HCl, pH 7.9). Subsequently, the resulting samples were collected and analyzed by sodium dodecyl sulfate polyacrylamide gel electrophoresis (SDS-PAGE).

### Enzyme Activity Assay of AoNagase

The activities of purified AoNagase with chitosan and colloidal chitin as substrates were assayed by the 3, 5-dinitrosalicylic acid (DNS) method (Miller 1959; Qin et al., 2018a). The reaction system consisted of 0.35 ml 1% substrate solution (w/v, pH 6.0) and 0.05 ml 1 μg/ml enzyme solution. The system was kept at 50°C for 10 min, after which the reaction was stopped by adding 600 μl DNS reagent and the subsequent boiling in a water bath for 10 min. Then the reaction system was rapidly cooled in an ice-bath and centrifuged at 12,000 rpm for 5 min at RT, the supernatant was collected and the absorbance was measured at 540 nm. One unit of enzymatic activity of is defined as the amount of AoNagase required to release 1 μmol of D-glucosamine (GlcN) or GlcNAc per minute under the assay conditions. D-glucosamine hydrochloride and GlcNAc were used as standards, respectively.

The activity of purified AoNagase with the substrate of *p*NP-GlcNAc was assayed by the method of *p*NPG (Yang et al., 2014). A 400 μL reaction mixture containing 100 μl of 1 μg/ml enzyme solution, 200 μl of 2 mM *p*NP-GlcNAc solution and 100 μl of 200 mM sodium acetate buffer (pH 6.0) was incubated at 50°C for 10 min. The reaction was terminated by the addition of 400 μL 0.5 M NaOH solution, and then the absorbance of the reaction mixture was measured at 410 nm. One unit enzyme activity of AoNagase was defined as the amount of enzyme required to release 1 μmol of *p*NP per minute under the assay conditions.

The protein concentration was determined using the Bradford method (Lowry et al., 1951; Yi et al., 2018) using bovine serum albumin (BSA) as standard.

### Biochemical Characterization of AoNagase

The optimal pH for AoNagase was determined by measuring its enzymatic activity in different buffers (20 mM): sodium acetate buffer (pH 3.0–6.0), phosphate buffer (pH 6.0–8.0) and Gly NaOH buffer (pH 8.0–11.0). To determine pH stability, the enzyme solution was incubated in the buffers aforementioned at 25°C for 30 min, and the residual activities were determined by DNS method. To determine optimal temperature, the enzyme activity was measured in the temperature range of 30–90°C in 20 mM sodium acetate buffer (pH 6.0). To determine its thermostability, the enzyme solution was incubated in 20 mM sodium acetate buffer (pH 6.0) at different temperatures (30–90°C) for 30 min, and the residual activities were determined by DNS method. In addition, the effects of metal ions (Mn^2+^, Co^2+^, Cu^2+^, Ba^2+^, Ca^2+^, K^+^, Zn^2+^, Ni^2+^, Mg^2+^) on AoNagase activity were investigated. A final concentration of 1 mM was used for the metal ions. The assay mixtures contained no metal ions were used as control. The established optimum pH and temperature were used when testing the effects of metal ions on the AoNagase activity. The degree of inhibition or stimulation of protease activity was determined after 30 min and expressed as a percentage of control.

### Substrate Specificity and Kinetic Parameters of AoNagase

To exploit the substrate specificity of AoNagase, 1% (w/v) of various substrates including chitosan, colloidal chitin, carboxyl methyl cellulose (CMC) and cellobiose were incubated with enzyme solution under assay conditions for 24 h. The hydrolytic products were detected by thin-layer chromatography (TLC). Samples were spotted onto a silica gel plate (Merck, Darmstadt, Germany), using a mixture of isopropanol: water: ammonium hydroxide (15:1:7.5, v/v/v) as developing solvent, and a mixture of alcohol: p-anisaldehyde: sulfuric acid: acetic acid (89:5:5:1, v/v/v/v) as color-developing agent. The plates were dried with hairdryer and heated at 130°C in an oven for 3–4 min until the hydrolysates were visualized. In addition, 2 mM *p*NP-GlcNAc was incubated with enzyme solution and assayed by the method of *p*NPG.

The kinetic parameters of AoNagase were determined by assaying the enzyme activity using a range of substrate concentrations of chitosan (0.05%–2%, w/v) and colloidal chitin (0.05%–1%, w/v) under the optimal conditions determined previously. The kinetic constants *K*_*m*_ and *V*_*max*_ values were calculated by nonlinear regression using Origin software 2018 (Version 8.6, OriginLab Corp, United States) with the Enzyme Kinetics Module.

### Hydrolytic Patterns of AoNagase

To investigate the hydrolytic products of AoNagase, the purified enzyme (1.5 U/ml) was added to 1% (w/v, 100 mM sodium acetate buffer, pH 6.0) chitosan and a series of COS monomers (DP 2–6) solutions. The reactions were kept at 50°C for 5, 10, 15, 30, 60, 90, 120, 240 min and 24 h. The other reaction composed of purified enzyme (1.5 U/ml) and 1% colloidal chitin was kept at 50°C for 4, 12, 24, and 48 h. The reactions were immediately stopped by boiling in a water bath for 10 min. TLC was used to analyze hydrolysis products.

## Results

### Sequence Analysis of AoNagase

**FIGURE 1:**
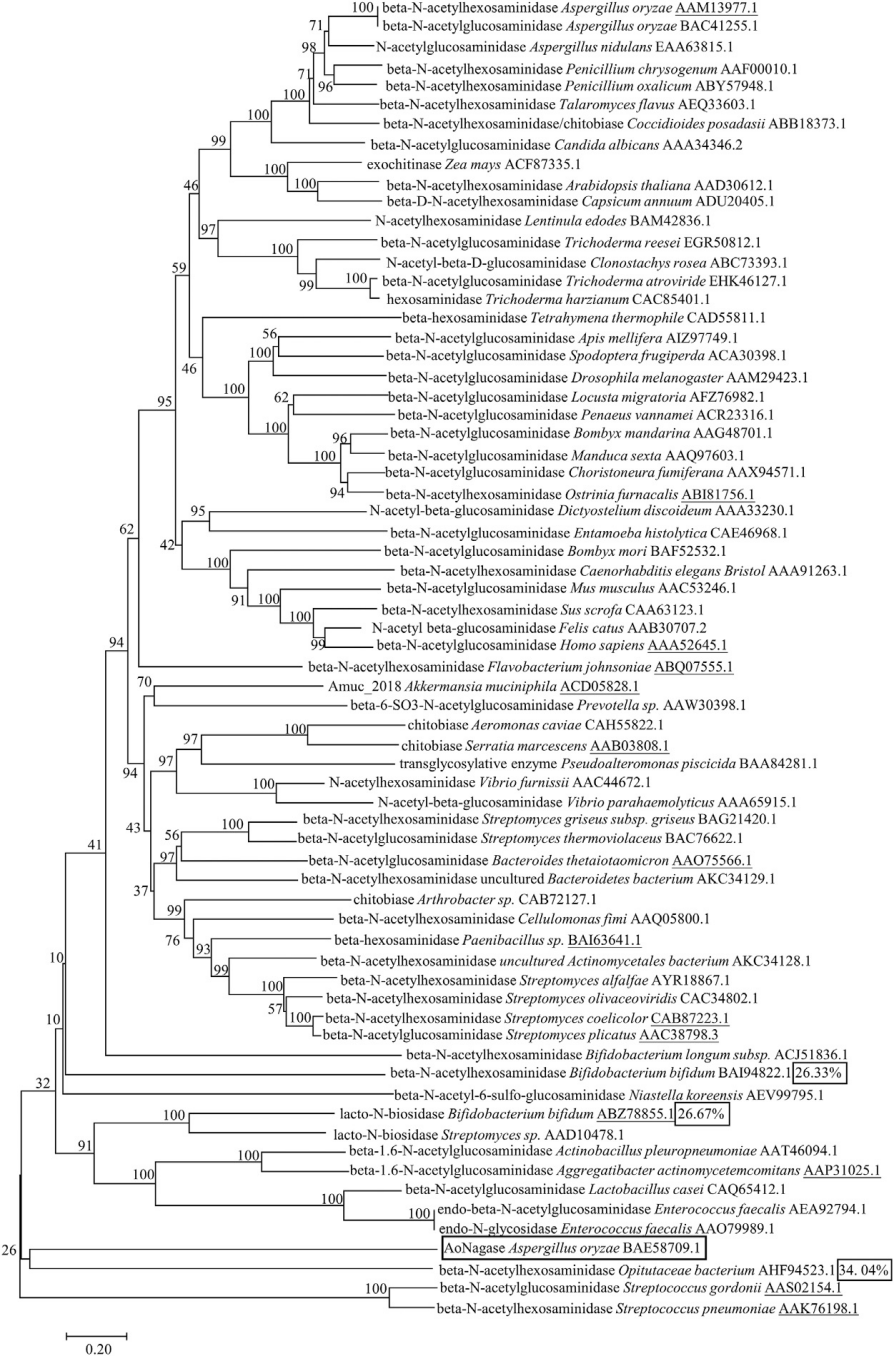
Phylogenetic analysis of AoNagase with 67 proteins from GH family 20 in the CAZy databases. Neighbor-joining tree shows phylogenetic relationships between AoNagase and other GH family 20 members from Uniprot and Protein Date Bank entries. All sequences from GH family 20 were labeled with names and GenBank accession numbers. The underlines indicate the proteins with known 3D structure.

An evolutionary tree of 67 protein sequences from GH family 20 was generated using neighbor-joining method to display their similarity with AoNagase (Figure 1). Interestingly, AoNagase shaped a deeply branched cluster in the evolutionary tree, indicating that it is genetically distinct from other reported β-N-acetylhexosaminidases in GH family 20. Furthermore, homology search by the BLASTp program revealed that AoNagase shared low identities with all known GH family 20 β-N-acetylhexosaminidases. AoNagase showed the highest sequence identity of 34.04%, 26.67% and 26.33% with the β-N-acetylhexosaminidases from *Opitutaceae bacterium* TAV5 (AHF94523.1) (Kotak et al., 2015), *Bifidobacterium bifidum* JCM 1254 (ABZ78855.1) (Ito et al., 2013) and *Bifidobacterium bifidum* JCM 1254 (BAI94822.1) (Miwa et al., 2010), respectively (Figure 1, box marked). The low homology indicated that AoNagase should hardly have similar functions to these proteins in GH family 20. We tried to use SWISS-MODEL Repository to generate 3D protein structure models and evaluate the resulting model (data not shown), but the target-template sequence identity (23.33%), the global model quality estimation (0.60) and the quaternary structure quality estimation (−5.76) all failed to meet the standard to predict the protein function (Guex et al., 2009; Studer et al., 2020). Thus, AoNagase may have a different fold compared with other GH 20 family members.

### Gene Cloning, Enzyme Expression and Purification

**FIGURE 2:**
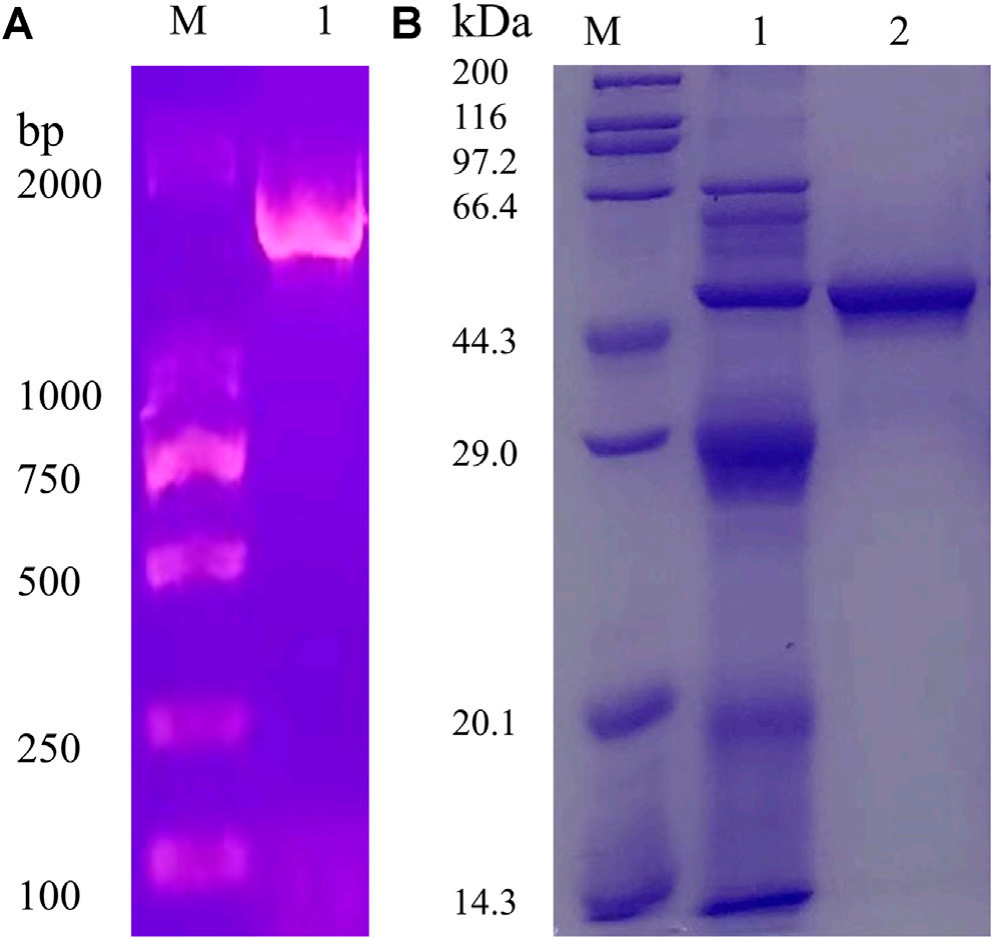
The agarose gel electrophoresis (1%) of the PCR products **(A)**. The SDS-PAGE (12%) of the proteins during purification of the recombinant AoNagase expressed in *P. pastoris* **(B)**; Lane 1: The crude enzyme in the supernatant of lysates; Lane 2: The purified enzyme, eluted by 350 mM–400 mM NaCl solution.

The full-length AoNagase gene had an open reading frame (ORF) of 1554 bp, encoding 517 amino acids. The PCR product was obtained using *A. oryzae* cDNA as template (Figure 2A). The attempts to express aonagase in *E. coli* expression system ended up with insoluble intracellular inclusion body (data not shown). Therefore, we turned to express AoNagase in *P. pastoris*. The recombinant protein was purified by Q-Sepharose Fast Flow ion exchange column and the purification table was shown in Supplementary Table 1.

As shown in Figure 2B, the purified recombinant AoNagase migrated as a single band on SDS-PAGE with a molecular mass of around 48 kDa, which was smaller than the predicted molecular weight (57.57 kDa). Sequence analysis revealed that there is a possible serine proteinase Kex2 (a signal peptidase participates in yeast protein secretory), recognizing site between amino acid 75 and 76, which may result in the 1–75 amino acid residues of AoNagase being cleaved after protein expression (Wu et al., 2013; Kim et al., 2015). Without the N-terminal 1–75 amino acid residues, the theoretical molecular mass of the resultant recombinant protein is 48.77 kDa, which was in good accordance with the result in Figure 2B.

### Biochemical Characterization of AoNagase

**FIGURE 3:**
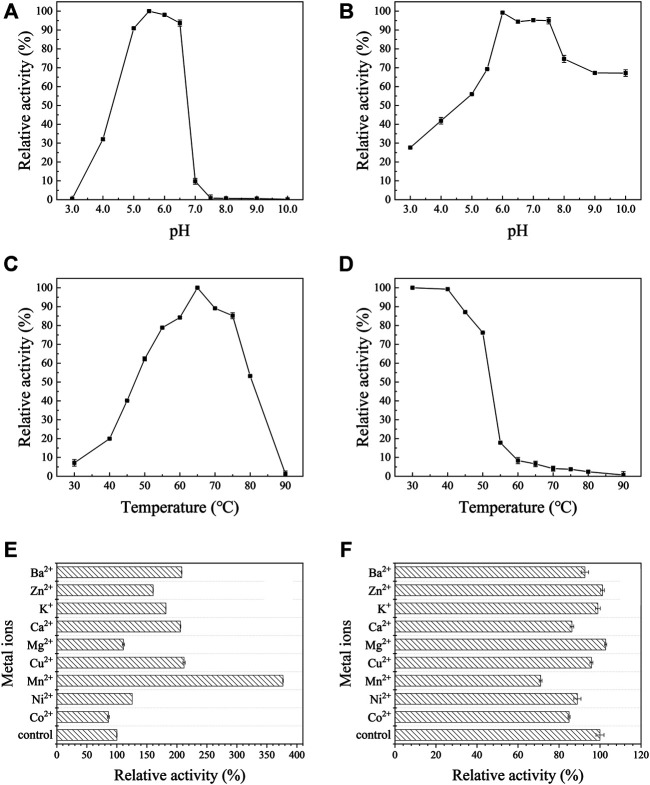
The biochemical characterization of AoNagase, optimal pH **(A)**, pH stability **(B)**, optimal temperature **(C)**, thermostability **(D)** and the effects of metal ions on AoNagase activity with chitosan **(E)** and colloidal chitin **(F)** as substrat. The results were the average of 3 sets of parallel samples, and the standard deviation (SD) was taken as the error bar.

Effects of pH, temperature and metal ions on the activity of AoNagase were examined. The purified AoNagase was active at a range of pH from 4.0 to 8.0 with an optimum pH at 5.5 (Figure 3A). It retained over 90% of its activity between pH 5.0–6.5. The enzyme exhibited good pH stability within the range of 6.0–7.5, retaining over 94% activity after pre-incubated at 25°C for 30 min (Figure 3B). AoNagase was active at a wide range of temperature with an optimum temperature at 65°C (Figure 3C). The enzyme was stable up to 50°C, with more than 75% activity remaining after incubated for 30 min (Figure 3D). Considering the thermal stability, the reaction temperature should be lower than 50°C. Taken together, in order to ensure the stability of AoNagase, the reaction condition was set at pH 6.0 and 50°C.

Metal ions may influence the enzyme activity by interacting with disulfide bonds to change the structure of the enzyme or reacting with the substrate (Zheng et al., 2019). Therefore, the effects of metal ions on AoNagase activity were tested. As shown in Figure 3E,F, metal ions had different effects on the activity of AoNagase when hydrolyzing chitosan and colloidal chitin. When using chitosan as substrate, the activity of AoNagase was strongly affected by the tested metal ions (Figure 3E). At the concentration of 1 mM, Mn^2+^ had the greatest stimulating effect (370% of the original activity); Cu^2+^, Ca^2+^, Ba^2+^, K^+^, Zn^2+^ and Ni^2+^ had evident promoting effects on the AoNagase activity; while Mg^2+^ and Co^2+^ hardly affected the AoNagase activity. When the colloidal chitin was used as substrate, AoNagase activity was slightly inhibited by most tested metal ions, of which Mn^2+^ was found to be the strongest inhibitor (70% of the original activity) (Figure 3F).

### Substrate Specificity and Kinetic Parameters of AoNagase

The purified AoNagase was further tested for its capability to hydrolyze different β-1,4 linked substrates. AoNagase showed the highest activity towards chitosan (508.26 U/mg), followed by *p*NP-GlcNAc (32.07 U/mg) and colloidal chitin (29.78 U/mg), respectively (Supplementary Figure 1A). As shown in Supplementary Figure 1B, after hydrolyzed for 24 h, the major products of chitosan were chitobiose [(GlcN)_2_], chitotriose [(GlcN)_3_] and a little GlcN, the major products of colloidal chitin were GlcNAc and N-acetyl chitobiose [(GlcNAC)_2_]. The product of colloidal chitin (Lane 2) was much less visible than that of chitosan (Lane 1) when the same volume of hydrolysis product was loaded, indicating that the enzyme is less active in hydrolyzing colloidal chitin. When the loading volume was increased by 4 times (Lane 3), the products of colloidal chitin were clearly visible (Supplementary Figure 1B). The Michaelis-Menten constants of AoNagase for chitosan and colloidal chitin were determined. The kinetic parameters (*K*_*m*_ and *V*_*max*_) of AoNagase were determined to be 1.51 mM, 1106.02 U/mg for chitosan and 0.41 mM, 40.31 U/mg for colloidal chitin (Supplementary Figure 3). AoNagase had no activity towards (CMC) and cellobiose (data not shown).

### Hydrolysis Patterns of AoNagase

**FIGURE 4:**
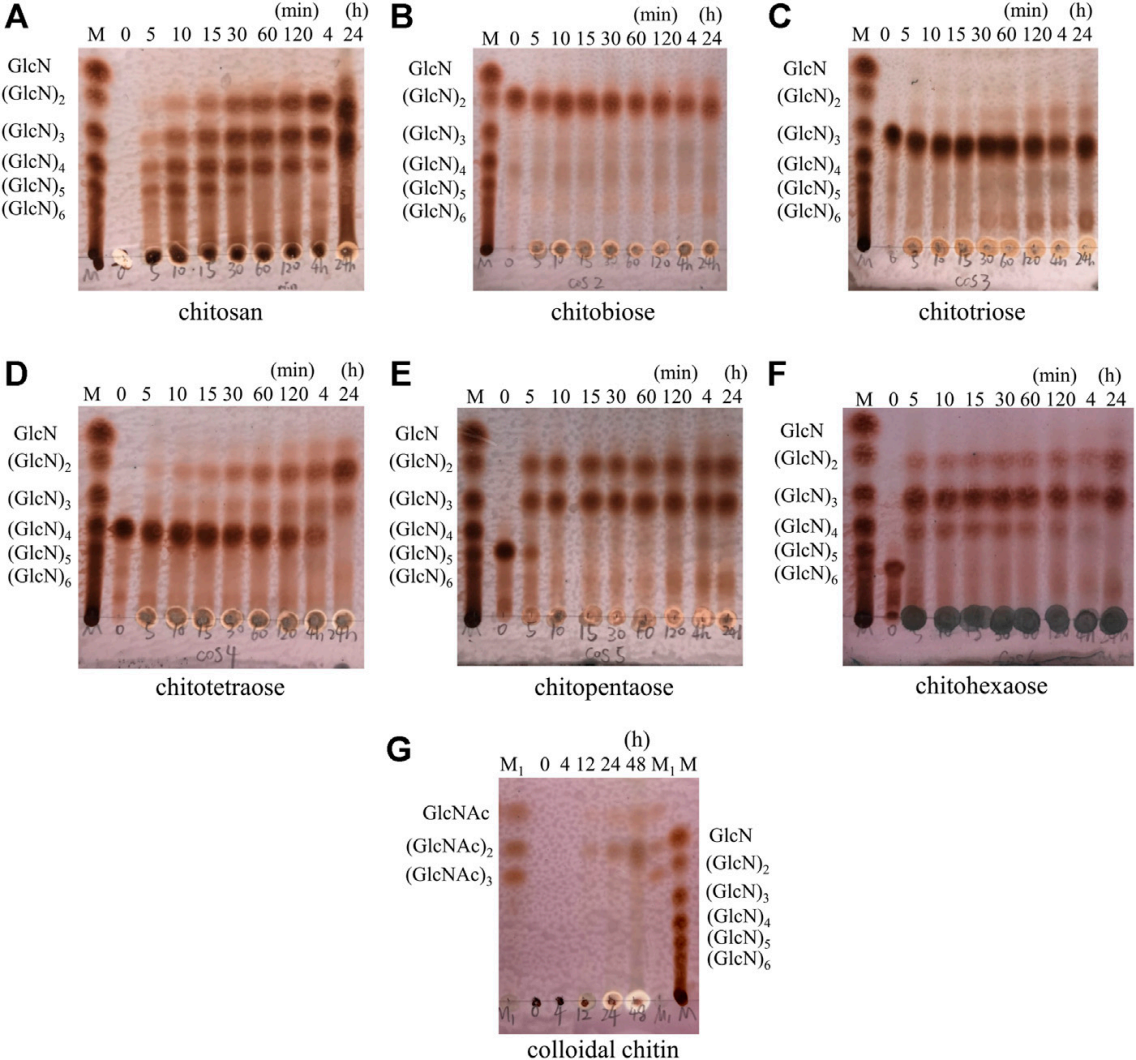
Hydrolytic process of AoNagase toward chitosan, COS (DP 2–6) and colloidal chitin by TLC. The substrates were chitosan **(A)** (GlcN)_2_ **(B)** (GlcN)_3_ **(C)** (GlcN)_4_ **(D)** (GlcN)_5_ **(E)** (GlcN)_6_ **(F)** and colloidal chitin **(G)**. Lane M (GlcN)_1–6_; Lane M_1_ (GlcNAc)_1–3_.

The hydrolysis progress of chitosan, COS monomers (DP 2–6) and colloidal chitin by AoNagase was examined and the hydrolysis products were analyzed by TLC (Figure 4). When chitosan was used as substrate (Figure 4A), its hydrolysis resulted in a series of COS with DP from two to six within 4 h. The DP of COS products changed with the reaction time. After prolonging reaction time to 24 h, GlcN was yielded.

The hydrolytic properties of AoNagase toward COS (DP 2–6) were also investigated to further elucidate the hydrolysis pattern of AoNagase. As shown in Figure 4B,C, AoNagase could cleave the β-1,4 linkage in [(GlcN)_2_] and [(GlcN)_3_] after prolonged incubation for 24 h, but the hydrolysis capacity was low. Nevertheless, AoNagase could efficiently hydrolyze chitotetraose [(GlcN)_4_], chitopentaose [(GlcN)_5_] and chitohexaose [(GlcN)_6_], ending up with the final hydrolysis products of GlcN, [(GlcN)_2_], and [(GlcN)_3_] (Figure 4D–F), which were the same as the end-products of chitosan (Figure 4A).

When colloidal chitin was used as substrate, TLC showed that no oligosaccharides were detected during the first 4 h, a small amount of GlcNAc and [(GlcNAc)_2_] could be detected after 12 h and the oligosaccharides accumulated with reaction time increase (Figure 4G). According to the hydrolysis property, AoNagase is an endo-type GH family 20 β-N-acetylhexosaminidase.

## Discussion

In nature, *A. oryzae* is able to hydrolyze and utilize chitin and chitosan to satisfy its physiological needs. Genome sequencing analysis (GenBank: PRJNA20809) further confirmed that the *A. oryzae* genome contains a diverse array of genes encoding β-N-acetylhexosaminidases (Machida et al., 2005; Cerqueira et al., 2014). Therefore, it is feasible to identify a novel β-N-acetylhexosaminidase from *A. oryzae.*

The phylogenesis analysis among AoNagase and other GH family 20 protein sequences demonstrated that AoNagase is a novel member of GH family 20 β-N-acetylhexosaminidases (Figure 1). The molecule weight of AoNagase expressed in *P. pastoris* was shorter than its theoretical value (Figure 2B). Sequence analysis showed that there is a possible Kex2 recognizing site between amino acid 75 and 76 of AoNagase and the molecule weight of truncated AoNagase (76–517 amino acid) was in good accordance with that showed in Figure 2B. The N-terminal truncation of AoNagase did not affect the enzymatic properties (Kim et al., 2015). Regarding the optimum pH and temperature, 85% of β-N-acetylhexosaminidases have an optimal pH in the range of pH 5.0–8.0 (Zhang et al., 2018). The optimal pH for AoNagase (pH 5.5) is within this range. Besides, 85% of β-N-acetylhexosaminidases have an optimum temperature in the range of 37–60°C, with nearly 60% of them in the range of 37–50°C (Chen et al., 2015). However, the optimum temperature of AoNagase is 65°C, which is slightly higher than that of most β-N-acetylhexosaminidases. Research shows that metal ions such as Cu^2+^, Hg^2+^ and Co^2+^ had inhibitory effects on most of the chitinases and chitosanases, while metal ions such as Mg^2+^, Ca^2+^ and Mn^2+^ had stimulating effects (Wang et al., 2008; Sosnowska et al., 2018). Interestingly, Mn^2+^ promotes the hydrolysis of chitosan by AoNagase, but inhibits the hydrolysis of chitin. In addition, AoNagase differs from other GH family 20 β-N-acetylhexosaminidases in terms of substrate specificity and hydrolytic pattern.

At present, many substrates have been reported to be degraded by the β-N-acetylhexosaminidases in GH family 20. A GH family 20 β-N-acetylhexosaminidase from *Trichoderma reesei* only uses *p*NP-GlcNAc as its substrate (Chen et al., 2015). Two novel GH family 20 β-N-acetylhexosaminidases from *Paenibacillus sp*., have the ability to hydrolyze glycosphingolipids, *p*NP-GalNAc, *p*NP-GlcNAc and to degrade ChOS (DP 2–3) into GlcNAc (Sumida et al., 2009). A β-N-acetylhexosaminidase from the fruiting body of *Lentinula edodes* (shiitake mushroom) can hydrolyze *p*NP-GlcNAc, *p*NP-GalNAc, it can also degrade ChOS (DP 2–6), colloidal chitin (46.3 U/mg) and mechanochemically ground chitin (39.9 U/mg) into GlcNAc (Konno et al., 2012). In this study, AoNagase was found to be capable of hydrolyzing not only *p*NP-GlcNAc and colloidal chitin, but also chitosan. In spite of so many substrates have been reported to be degraded by β-N-acetylhexosaminidases in GH family 20, it is the first time an enzyme in GH family 20 was reported to be able to hydrolyze chitosan.

There is a conserved amino acid residue (H/N-X-A/C/G/M-**D-E**-A/I/L/V) at the catalytic sites of the β-N-acetylhexosaminidases in GH family 20 (Gutternigg et al., 2007). Among them, aspartic acid (D) and glutamic acid (E) are highly conserved, glutamic acid acts as a general acid/base residue for protonation, while aspartic acid acts to orient the C_2_-acetylamino group to the correct position for nucleophilic attack of the water molecule (Lemieux et al., 2006; Zhang et al., 2018). The conserved amino acid sequence in AoNagase is H-I-G-A-**D**-**E**-Y-D-K-D-L-V, the highly conserved active center -D-E- ensures that AoNagase possesses the activity of a β-N-acetylhexosaminidase to hydrolyze colloidal chitin and *p*NP-GlcNAc. The mechanism for AoNagase to hydrolyze chitosan and COS (DP 2–6) requires further experimental analysis and mechanistic studies. The lack of structural information and the implausibility of the simulated structure limited further molecular designing studies about AoNagase. The discovery of AoNagase will hopefully yield new insights into substrate binding and the catalytic mechanisms of β-N-acetylhexosaminidases of GH family 20.

## Conclusion

In conclusion, a novel gene AoNagase from *A. oryzae* was cloned and successfully expressed in *Pichia pastoris*. The biochemically characterization of AoNagase revealed that it exhibited favorable thermostability and pH stability. This is the first time a β-N-acetylhexosaminidase in GH family 20 was found to effectively degrade chitosan and colloidal chitin. Mn^2+^ significantly promoted the hydrolysis of chitosan by AoNagase, but inhibited the hydrolysis of chitin. The kinetic parameters indicated that AoNagase had high affinity for the substrates. The investigations of AoNagase will be helpful for the further understanding of the characteristics of GH family 20, and will provide a theoretical basis for the enzymatic conversion of chitinous biomass into aminooligosaccharides.

## Data Availability

The datasets presented in this study can be found in online repositories. The names of the repository/repositories and accession number(s) can be found below: https://www.ncbi.nlm.nih.gov/genbank/, BAE58709.1.
