# Supplementary material for: A Novel GH Family 20 β-N-acetylhexosaminidase With Both Chitosanase and Chitinase Activity From Aspergillus oryzae
Source: Front Mol Biosci. 2021 May 19;8:684086. doi: 10.3389/fmolb.2021.684086 (PMC8170477; doi:10.3389/fmolb.2021.684086)
Supplement: Supplementary file 1 [file DataSheet1.docx]

Supplementary Material


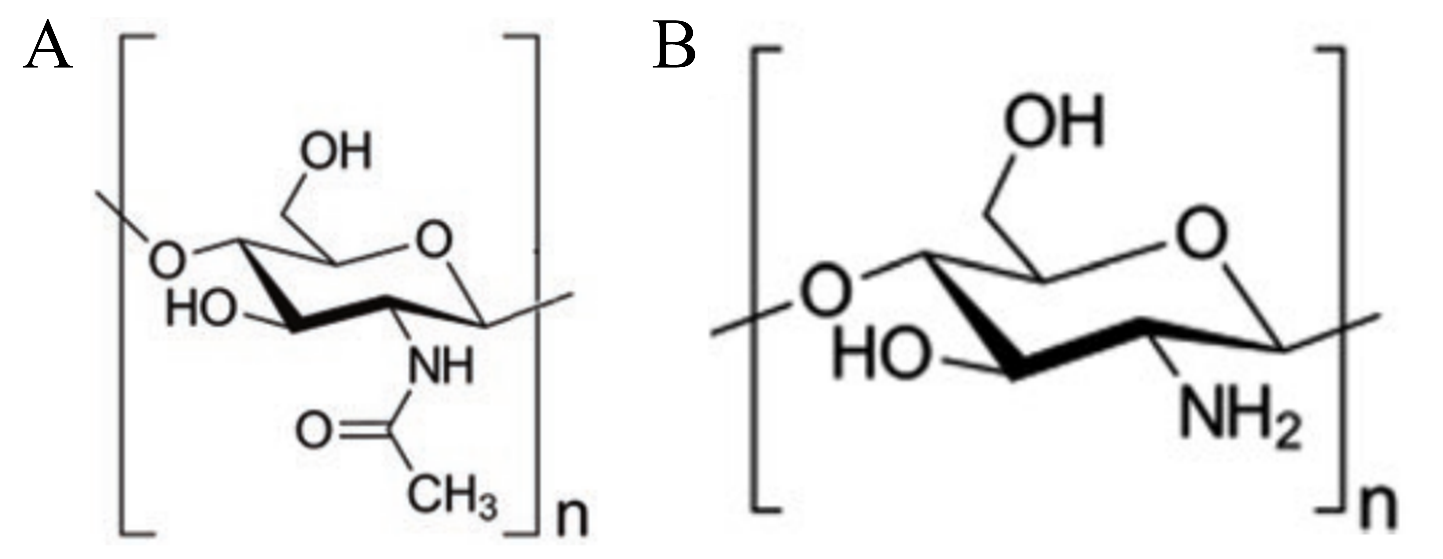


**Supplementary Figure 1.** The structures of chitin (A) and chitosan (B).


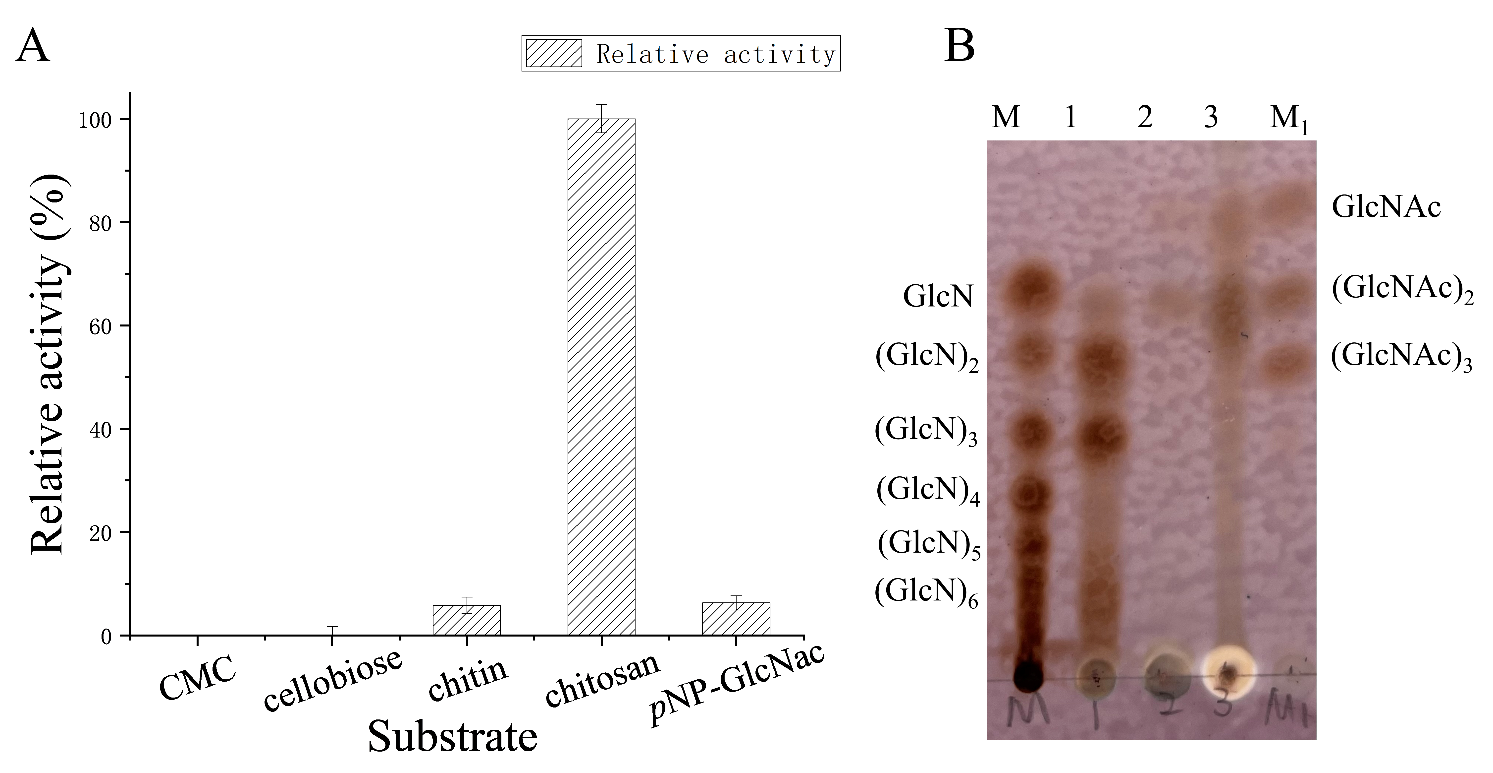


**Supplementary Figure 2.** The relative enzyme activity of AoNagase with different substrates (A) and the hydrolysis products of AoNagase (B). The hydrolysis products of chitosan (Lane 1) and colloidal chitin (Lane 2) with the same volume of hydrolysis products loaded; Lane 3: The hydrolysis products of colloidal chitin loaded with 4 times of the volume in Lane 2; Lane M: (GlcN)_1-6_; Lane M_1_: (GlcNAc)_1-3_. The results of Figure 2A were the average of 3 sets of parallel samples, and the standard deviation (SD) was taken as the error bar.

**
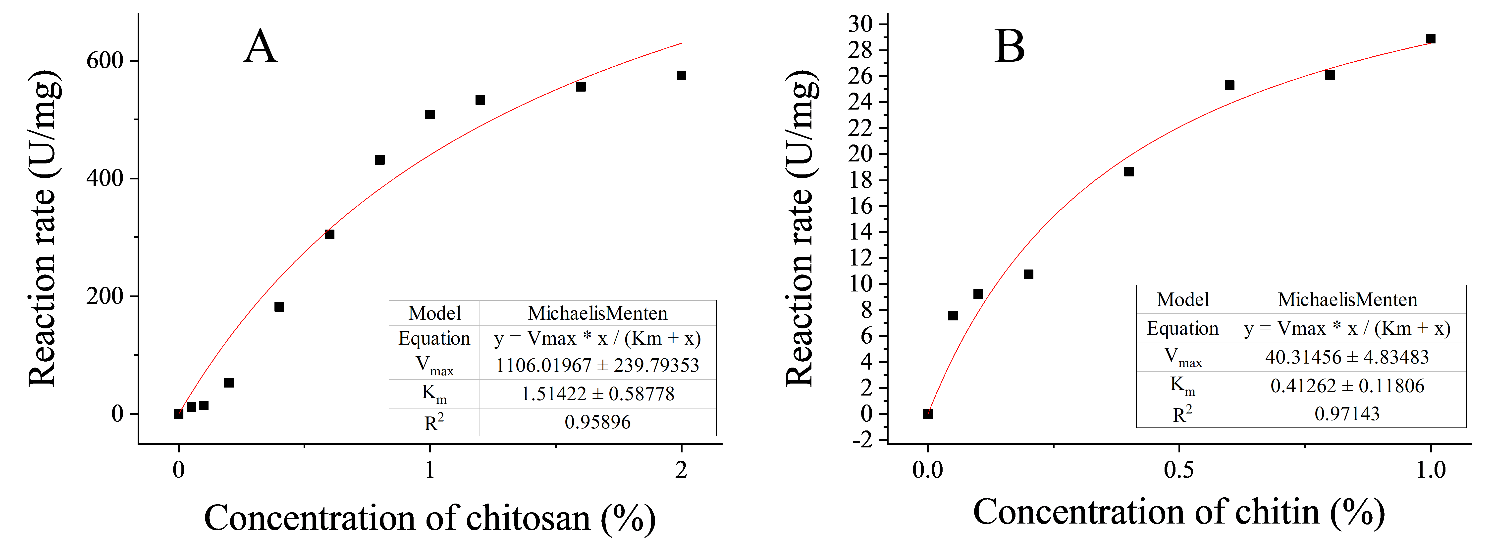
**

**Supplementary Figure 3.** The Michaelis-Menten equation plots for chitosan (A) and colloidal chitin (B) as substrate. Data represent the means of three independent experiments (n = 3).

**Supplementary Table 1.** Purification table of AoNagase

| Purification step | Total activity (U) | Protein (mg) | Specific activity (U/mg) | Purification factor (fold) | Recovery (%) |
| --- | --- | --- | --- | --- | --- |
| crude enzyme | 4348.13 | 28.18 | 154.30 | 1 | 100 |
| ion exchange | 2350.48 | 4.62 | 508.26 | 3.29 | 54.06 |

^*^ Data represent the means of three independent experiments (n = 3).

^**^ 100 mL BMMY culture medium was uesd. The yield of purification is 46.24 mg/L (mg recombinant protein per L of BMMY culture).

^***^ Chitosan was used as substrate. The purification factor and recovery with colloidal chitosan as substrate were similar to those with chitosan as substrate.
